# Modulating Renal Fibrosis: The Emerging Role of Hepatocyte Growth Factor

**DOI:** 10.1155/ijne/6821899

**Published:** 2026-08-25

**Authors:** Annalia Ferrer, Joshua Kim, Arleigh-Ann Byer, Mohamed Ahmed

**Affiliations:** ^1^ California Northstate University College of Medicine, 9700 West Taron Drive, Elk Grove California, 95758, USA, cnsu.edu

**Keywords:** diabetes, fibrogenic process, hepatic growth factor, hypertension, renal fibrosis

## Abstract

**Introduction and Rationale:**

Hepatocyte growth factor (HGF) is a multifunctional protein that is associated with cellular growth and expansion. HGF has been known to be heavily expressed in renal tissue and for its role in wound healing and tissue proliferation. This review article aimed to investigate the pathogenesis of HGF in renal fibrosis in addition to its relationship with interconnected inflammatory diseases such as hypertension and diabetes.

**Methods:**

Several online databases such as PubMed and Google Scholar were used up to 2025 to identify and compare studies that looked at the relationship between renal fibrosis and HGF in preclinical and clinical settings.

**Results and Conclusion:**

HGF was shown to reduce the progression of renal fibrosis through mechanisms such as transforming growth factor‐β (TGF‐β) antagonism, enhancement of endothelial repair, and promotion of extracellular matrix (ECM) degradation. The results indicate that HGF shows promising results on slowing the progression of renal fibrosis. However, the lack of studies using human models to research the antifibrotic effects of HGF as well as the associated risk of HGF in developing diabetes, insulin resistance, and hypertension calls for further investigations about future applications of HGF to treat renal fibrosis.

## 1. Introduction

The discovery of the mitogenic protein hepatocyte growth factor (HGF) in 1989 marked a significant milestone in the field of cellular biology, as it was quickly found to exhibit a variety of biological effects [1]. This multifaceted protein has since been extensively studied, revealing its critical roles in several physiological and pathological processes [2]. Its pleiotropic nature has been especially beneficial in understanding and managing conditions related to inflammation [3], cancer [4], and the process of new blood vessel formation known as angiogenesis [5]. One of the pivotal aspects of HGF’s action is its interaction with the c‐Met protein, a transmembrane receptor tyrosine kinase [6]. This receptor is unique in its ability to bind exclusively to HGF, and its upregulation has been associated with a worsened prognosis in patients suffering from renal cell carcinomas [7]. Studies in mouse models have shown that the vascular endothelial growth factor (VEGF) multityrosine kinase inhibitor Sunitinib can be resisted in scenarios of increased HGF expression [8], shedding light on HGF’s potential role in contributing to complex pathologies like renal fibrosis [9].

As HGF is heavily implicated in the pathogenesis of renal fibrosis in the setting of chronic renal disease, studies investigated the utilization of HGF in renal fibrosis [10]. HGF has been shown to lower creatinine levels and blood urea nitrogen levels and increase insulin clearance [11], factors that are used to assess current renal function in a setting of a renal disease process. Additionally, serum HGF was shown to be implicated in other inflammatory states, such as hypertension and type 2 diabetes [12]. Hypertension and type 2 diabetes are contributors to renal fibrosis through inflammatory injuries and oxidative stress that lead to renal injury [13]. Thus, these studies show a potential therapeutic and diagnostic value of HGF in renal fibrosis.

## 2. Methods

A comprehensive literature search for this narrative review was conducted using the PubMed/MEDLINE database and Google Scholar to identify articles about HGF. Search terms relevant to the topic of interest included: “hepatocyte growth factor,” “HGF,” “renal fibrosis,” “hypertension,” and “diabetes.” Terms were combined using AND/OR to help refine results.

Articles were considered for inclusion based on the following criteria:

English written original research articles, clinical practice guidelines, and case series that addressed the biological mechanism and applications of HGF, its clinical association with hypertension and diabetes, and its potential therapeutic role in renal fibrosis. No restrictions were placed on study design or publication date. Articles were excluded if they were identified as editorials, commentaries, and opinion pieces with no original data or relevance to HGF and its clinical applications in the context of renal fibrosis.

Titles and abstracts of retrieved records were screened for relevance during the initial study selection. Full‐text articles were then obtained for all potentially eligible studies and assessed against inclusion and exclusion criteria. Emphasis was placed on landmark studies with more recently published literature. Relevant data were extracted from studies that met the inclusion criteria, including study design, population characteristics, key findings, and clinical implications. Findings were organized thematically according to (1) biological mechanisms and signaling pathways of HGF; (2) the clinical association of HGF with hypertension and diabetes; and (3) the therapeutic potential in renal fibrosis.

Available evidence was then summarized through qualitative synthesis. Areas of consensus and gaps in current literature in reference to clinical applications were identified. As this article follows a narrative review design, no formal risk‐of‐bias assessment or meta‐analytic pooling was performed. This article does not adhere to PRISMA guidelines.

The methodological quality of this review was guided by the Scale for the Assessment of Narrative Review Articles (SANRA).

### 2.1. Renal Fibrosis

Renal fibrosis is the accumulation of scar tissue and extracellular matrix (ECM) deposits, which characterizes the late‐state manifestation of progressive chronic kidney disease (CKD) [14].

There are six stages of CKD, a staging that is dependent on multiple factors such as glomerular filtration rate (GFR), albuminuria, and present imaging or history of any renal disorders or abnormalities [15]. The first stage of CKD is G1, which is categorized by having a GFR of 90 mL/min/1.73 m^2^ and is followed by stages marked with progressively worse GFR all the way to G5, in which the GFR is 15 mL/min/1.73 m^2^ [15]. Fibrosis in late‐stage CKD is thought to be an intermediate to kidney dysfunction and decreased blood flow, which often leads to organ failure and the need for a kidney transplant seen in patients with G4 and G5 stages of CKD [16].

The mechanism behind renal fibrosis stems from uncontrolled healing processes in the body as a response to excessive kidney scarring, leading to the accumulation of renal ECM deposits and even organ failure [17]. Systemic issues, such as diabetes, hypertension, and autoimmune diseases, contribute to the pathology of the disease via increased glomerular pressure and destruction of glomerular cells, leading to the unbalanced wound‐healing response [18].

The irreversible damage of renal fibrosis stems from myofibroblasts that enhance the fibrotic process [19]. While transforming growth factor‐β1(TGF‐β1) is observed to be an affiliated factor of myofibroblasts, there is also the TGF‐β1/Smad2 and 3 pathway that results in the development of profibrotic components such as fibronectin and collagen [20]. Additionally, mesangial cells of the glomerulus can contribute to renal fibrosis through an abundance of ECM [21]. The hyperproliferation of mesangial cells in response to kidney injury leads to the increased expression of proinflammatory cytokines, matrix metalloproteinase (MMP) enzymes, and ECM proteins [22].

Most treatments for renal fibrosis currently aim to control the progression of the disease; however, there are no therapeutic options available to prevent or cure renal fibrosis [17]. Due to its renoprotective function, more studies have begun to investigate HGF and its possible role in reversing and controlling renal fibrosis [17].

#### 2.1.1. Relationship of HGF With Diabetic Kidney Disease (DKD) and Renal Fibrosis

HGF has also been implicated in metabolic disorders, such as insulin resistance, a condition that can contribute to the development of DKD and renal fibrosis [1].

In diabetes, a persistent hyperglycemic state increases plasma osmolarity, creating an osmotic pressure shift that enhances GFR as a compensatory mechanism [23]. This hyperfiltration upregulates tubular glucose reabsorption, partly through increased expression of transporters such as sodium–glucose cotransporter‐2 (SGLT‐2), which further worsens hyperfiltration and activates pathways like the renin–angiotensin–aldosterone system [24]. These proinflammatory signaling pathways contribute to the progression of DKD, characterized by elevated urinary albumin excretion—typically greater than 300 mg/day or a random albumin‐to‐creatinine ratio (ACR) exceeding 20 mg/mmol [25]. Over time, chronic hyperglycemia and persistent albuminuria lead to structural damage of the glomeruli, reduced renal function, and ultimately end‐stage renal disease (ESRD), marked by irreversible renal fibrosis [26].

HGF has been demonstrated to have a correlation with increasing insulin resistance. The observed HGF elevation suggests a compensatory response to metabolic stressors causing insulin resistance rather than presenting as a primary pathological driver [27]. The study demonstrated that the rise in levels of HGF occurred prior to the compensatory response associated with the associated insulin resistance in the mouse models. Furthermore, mouse studies conducted on diet‐induced obese rats demonstrated a dose‐dependent relationship for the increase in pancreatic beta cell mass. In the liver, reversal of beta cell hyperplasia was induced via blockage of the c‐Met–insulin receptor complex [27].

Recently, HGF has become a promising biomarker of insulin resistance, as its synthesis is increased in insulin resistance states, and its receptor displays a resemblance to that of the insulin receptor [1]. A study conducted by García‐Ocaña et al. showed that mice that had an overexpression of HGF had increased insulin secretion and elevated glucose tolerance than their control counterparts [28]. Another study showed a similar relationship between insulin and HGF [29]. A study conducted by Roccisana et al. showed that when the c‐Met receptor was knocked out in mice, there was impaired glucose‐driven insulin secretion as well as decreased glucose tolerance [29]. What was also notable was that pancreatic beta cell morphology or amount was unchanged during these experiments [29]. HGF’s role in type 2 diabetes mellitus is also reflected in a human cohort study. Bancks et al. saw that individuals with higher HGF levels had a significantly higher risk of developing type 2 diabetes mellitus. Specifically, each increase in HGF was associated with a greater likelihood of future diabetes [30].

These findings are suggestive that the HGF/c‐Met axis may influence the progression of DKD through its influence on glucose tolerance as well as beta cell function. However, the clinical significance of these results remains uncertain, as the role of HGF in this process, whether as a compensatory mediator or a driver of diabetic kidney dysfunction, has yet to be defined.

#### 2.1.2. Relationship of HGF and Hypertension

Hypertension is a major driver of CKD progression and ultimately renal fibrosis through several different mechanisms, including endothelial dysfunction, oxidative stress, and activation of the renin–angiotensin–aldosterone pathway (RAAS).

Activation of RAAS leads to the downstream promotion of several pathways, including mitogen‐activated protein kinases (MAPKs), which contribute to inflammation and ECM deposition [31]. Furthermore, the hypertensive state results in the conversion of fibroblasts into myofibroblasts, causing tubulointerstitial injury and progressive renal scarring [31]. With the overall stimulation of TGF‐β, continuous deposition of ECM, and production of oxidative stress through reactive oxygen species (ROS), CKD can ultimately progress to renal fibrosis [32].

HGF serves as a key biomarker that can be utilized to analyze the degree of progression for hypertension [33]. In cases where patients had pre‐existing conditions alongside hypertension, serum HGF concentrations were higher compared to those who had pre‐existing conditions without hypertension [33]. Additionally, exogenous HGF displayed inhibitory effects on angiotensin II, a key component in the RAAS pathway, in ⅚ nephrectomized rats, showing a possible renoprotective effect [34].

Overall findings suggest that serum HGF may serve as a potential clinical biomarker of hypertensive complications and display renoprotective effects through modulation of profibrotic pathways. However, current evidence supporting these claims is largely preclinical. In addition, although observational human studies suggest a correlation between HGF and hypertension and severity, a lack of human clinical trials is needed to determine its clinical utility as a biomarker of hypertensive renal disease.

### 2.2. Possible Mechanisms of Renal Fibrosis

#### 2.2.1. TGF‐β

The TGF‐β cytokine, along with its various forms, plays a crucial role in the healing process through fibroblast proliferation [35]. Its biosynthesis revolves around the TGF‐β monomer undergoing dimerization within the endoplasmic reticulum, appropriate cleavage mechanisms to formulate small latent complexes (SLCs), and activation molecules such as proteases and integrins to release TGF‐β from the SLCs [36]. The TGF‐β anchors within the ECM via LTBP (Latent TGF‐β binding protein) [37]. Upon activation by activating factors such as αvβ6 integrin, TGF‐β1 results in the transformation of fibroblasts into myofibroblasts, causing ECM remodeling and generating mechanical tension [38]. A particular example involves kidney fibrosis, where TGF‐β mediates this condition through two pathways: the noncanonical (MAP kinase, p38/JNK, PI3K/Akt, and RhoA GTPase) pathway and the canonical (TGF‐β/Smad) pathway [39]. From transcribing profibrotic components to inhibiting fibroblast apoptosis, these pathways work to result in the fibrotic state of the kidney [40].

#### 2.2.2. Connective Tissue Growth Factor (CTGF)

CTGF, also known as CCN2, is a secreted matricellular protein that modulates various signaling pathways [41]. It plays significant roles as a downstream effector of TGF‐β, including cell adhesion, migration, angiogenesis, myofibroblast activation, and ECM deposition and remodeling [41–43]. Specifically, CTGF has been found to interact with various molecules, including cytokines (TGF‐β and VEGF), ECM proteins (fibronectin and heparan sulfate proteoglycans), growth factors (IGF1 and BMPs), and cell surface receptors, and all contribute to the fibrotic process [43].

As a mediator of TGF‐β’s profibrotic effects, CTGF plays a critical role in promoting ECM protein production and deposition through direct stimulation of their synthesis [42, 43]. CTGF expression is seen elevated in fibrotic tissues from patients with systemic sclerosis, as it plays a central role in the persistent accumulation of ECM in fibrotic tissues [42, 43]. The elevated expression is associated with fibroblast activation and collagen overproduction, which are hallmarks of systemic sclerosis [42, 43]. Additionally, the level of CTGF expression correlated with the extent of fibrosis, suggesting that it is critically involved in disease progression and severity [42, 43]. The relation of CTGF to TGF‐β is also seen in SSc fibroblasts (fibroblasts derived from patients with systemic sclerosis) [42, 43]. In these fibroblasts, TGF‐β signaling is overactive, leading to sustained activity of CTGF [43].

The influence of CTGF on fibrosis is not limited to SSc, but elevated CTGF levels have been observed in kidney and liver diseases associated with fibrosis [42, 44]. Particularly in renal diseases, elevated expression of CTGF can be seen in tubular epithelial cells and interstitial fibroblasts in tubulointerstitial fibrosis [44]. In hepatic cells, including hepatocytes, biliary epithelial cells, and hepatic stellate cells (HSCs), CTGF promotes fibrogenic activities [45]. Increased levels of CTGF have been detected in the serum of patients with liver fibrosis, indicating its function in hepatic disease [42].

#### 2.2.3. Angiotensin II

Angiotensin II is involved with RAAS and contributes to inflammation, proliferation, and thickening through the activation of the signal transducer and activator of transcription 3 (STAT3) pathway [46]. The STAT3 pathway is described as angiotensin II binding to fibroblast growth factor receptor 1 (FGFR1), which leads to the activation of STAT3 [46]. This contributes to the enhancement of TGF‐β, leading to collagen type 1 (Col‐1) buildup and development of renal fibrosis [47]. In addition to these pathways, this peptide hormone exerts fibrogenic effects through receptors such as toll‐like receptor 4 (TLR4) and FGFR1 in renal epithelial cells [46, 48]. The generation of inflammatory molecules such as TNF‐α, IL‐6, and MCP‐1 factors into the proinflammatory state, leading to tissue damage and advanced fibrosis in organs such as the kidney [49]. These components exemplify angiotensin II’s ability to mechanically stimulate fibrogenic proliferation in numerous manners, underscoring its multifaceted role.

#### 2.2.4. Platelet‐Derived Growth Factor (PDGF)

PDGF is known to drive wound healing and has been implicated in the progression of fibrosis in the kidneys and other organs [50]. The PDGF family is made up of five isomers, PDGF‐AA, PDGF‐AB, PDGF‐BB, PDGF‐CC, and PDGF‐DD, with two corresponding tyrosine kinases, PDGFR‐α and PDGFR‐β [51]. While normally different isomers are expressed in different locations in the kidney in rat models, studies have shown that all PDGF isomers are upregulated during times of kidney injury [52]. This leads to activation of profibrotic processes [52]. A study conducted by Floege et al. saw that transfusion of PDGF‐BB led to an increase in renal tubulointerstitial cell fibrosis and proliferation [53]. Suppression of this pathway is also observed to yield a protective factor against fibrosis [54]. In a study using mouse models, inhibition of PDGF‐CC was shown to reduce markers of renal inflammation and tubulointerstitial fibrosis compared to control models [54].

In human studies, Gesualdo et al. examined the PDGF‐β receptor (PDGFR‐β) role in pathologic and normal human kidneys [55]. They found that pathologic kidneys exhibit increased expression of PDGFR‐β, particularly in the glomeruli and interstitial tissue, compared to normal kidney samples [55]. These findings suggest that increased PDGFR‐β expression is associated with kidney disease progression and may contribute to the development and progression of renal fibrosis [55].

#### 2.2.5. Endothelin‐1

Endothelin‐1 belongs to a peptide family of potent vasoconstrictors, and abnormal activation leads to progression of CKD [56]. It is produced by renal tubular cells and mainly exerts its effects via two G‐protein–coupled receptor subtypes in the kidney, which contribute to the process, ET‐A and ET‐B [56]. Activation of ET‐A results in damage to the renal parenchyma and increases permeability of the glomerulus to albumin [56]. Stimulation of inflammation and fibrosis occurs via stimulation of lesions in the mesangium and podocytes, which leads to the loss of the negative charge barrier [57]. Furthermore, persistent vasoconstriction results in inflammation that is demonstrated as proteinuria on urinalysis and progression to glomerulosclerosis [58]. Opposingly, the role of ET‐B is thought to be protective and exhibits profibrotic activity when it is inhibited [59]. When activated, these receptors facilitate vasodilation, which promotes natriuresis and helps to clear ET‐1 from circulation [60].

#### 2.2.6. Plasminogen Activator Inhibitor‐1 (PAI‐1)

PAI‐1 comes from the serine protease inhibitor family [61]. Its primary role in the body is to inhibit the activity of tissue‐type plasmin activator and urokinase, thereby inhibiting fibrinolysis [62]. In diseased and aging kidneys, it is highly expressed in the tubules, interstitium, and podocytes [63]. During this upregulated state, it works in tandem with multiple other proteins to contribute to fibrotic damage and cell death of the renal epithelium [64]. A 2021 study found that cells with high expression of PAI‐1 are associated with decreased levels of an anti‐aging protein, klotho [63]. This results in downstream epithelial dysfunction and plasticity via upregulation of profibrotic cytokines like TGFβ [65]. In addition, this results in sustained p53 signaling, which leads to cell cycle inhibition and apoptosis through impairment of renal epithelial regeneration [66].

#### 2.2.7. Interleukins

Several proinflammatory cytokines, specifically interleukins, have been implicated in the pathophysiology of renal fibrosis. One of the best‐studied inflammatory biomarkers, IL‐6, is believed to be implicated in damage to renal parenchyma as it leads to elevated GFR and albuminuria through the modification of renal hemodynamics [67]. High levels of IL‐6 have been shown to correlate with increased progression of CKD [68]. IL‐1β and IL‐18 mediate inflammation in CKD after being cleaved into active forms by the NLRP3 inflammasome [68]. Thereafter, they both play active roles in calcification and fibrosis of vascular tissue [68]. Studies performed on mice demonstrated benefit for the reduction of kidney disease progression by inhibiting the cleavage of IL‐1β and IL‐18 [68]. In addition, IL‐33 appears to promote renal fibrosis through multiple, interconnected mechanisms, through binding to the ST2L receptor, triggering and recruitment of inflammatory mediators, and promotion of fibroblast proliferation and ECM deposition [69].

Conversely, anti‐inflammatory interleukins play a crucial role in halting the progression of inflammation in order to prevent further damage to the surrounding tissue [70]. In addition, the actions of these interleukins promote wound healing and angiogenesis [70]. Among these, IL‐10 has shown to have immunosuppressive properties that may attenuate fibrosis [71]. A study conducted on unilateral ureteral obstruction (UUO) saw that IL‐10 knockout mice exhibited higher levels of inflammatory cell infiltration and proinflammatory cytokines as well as increased renal tubulointerstitial collagen deposition, which together promote renal fibrosis [71]. These findings suggest that IL‐10 may play a protective role against renal fibrosis.

#### 2.2.8. HGF Compared to the Other Molecules

While HGF is noted for its significant role in regeneration, it is observed to hold a crucial impact when it comes to renal fibrosis [72]. The fibrogenic process consists of multiple variables, one of which involves TGF‐β1 [9]. HGF demonstrated its ability to sequester the transcription of TGF‐β1 genes by effectively disrupting the Smad signal transduction affiliated with TGF‐β1 [9]. In addition to its involvement with TGF‐β1, HGF further limits the progression of kidney dysfunctionality through its impact on myofibroblast formation and PDGF [10].

Additionally, in comparison with other known researched antifibrotic agents used in renal fibrosis research, such as bone morphogenetic protein‐7 (BMP‐7), HGF showed similar efficacy in suppressing tubulointerstitial fibrosis and preserving renal function in the setting of different renal inflammatory processes, albeit through different processes [73]. HGF is shown to have synergistic effects with other renal‐protective treatments such as anti‐angiotensin 2 therapies, showing an impressive decrease in renal fibrotic lesions [74]. While HGF shows impressive antifibrotic properties in animal and in vitro studies, its effects are still yet to be seen in human studies [74].

To date, no human trial has successfully tested HGF in either established renal fibrosis or CKD progression. Despite robust preclinical studies, there have been several challenges that have limited translation to human renal fibrosis therapy, one of which is the oncogenic risk. The HGF receptor is a tyrosine kinase that can be involved in tumor progression and metastasis through the activation of the HGF/c‐MET pathway, raising safety concerns for long‐term supplementation, particularly in populations on high‐dose immunosuppressive therapy [75].

For human studies involving HGF as a therapeutic agent, strategies to avoid the induction of tumorigenesis would first have to be developed [11].

In addition, delivery of systemic HGF poses another challenge due to its relatively short life and pleiotropic effects [76].

There has been proven efficacy of engineered solutions via natural polymer nanoparticles, demonstrating improved targeting in rodent models, but none have entered clinical trials [77].

#### 2.2.9. Analysis of Impact of HGF on Pathways and Factors Involved in Renal Fibrosis

**FIGURE 1:**
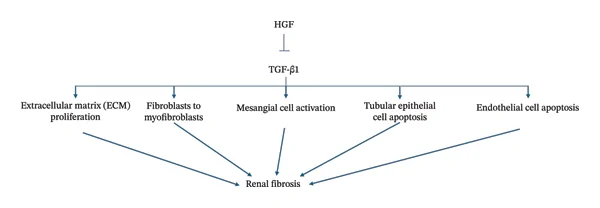
HGF inhibition of TGF‐β1–mediated renal fibrosis. TGF‐β1 promotes renal fibrosis through increased ECM synthesis and deposition, leading to increased collagen and fibronectin production, myofibroblast differentiation, mesangial cell activation, and tubular epithelial and endothelial cell apoptosis. These processes result in interstitial fibrosis and glomerulosclerosis. HGF plays a role in inhibiting TGF‐β1–mediated fibrotic processes [9].

HGF has already been demonstrated to significantly attenuate renal fibrosis through multiple pathways and mechanisms, as illustrated in Figure 1 [9, 78]. One of the key relationships that HGF has influence over is the TGF‐β pathway [79]. TGF‐β has been well established as a major contributor to the progression of renal fibrosis through promotion of the synthesis of ECM components and the subsequent inhibition of ECM degradation [80]. This accumulation of ECM leads to the development of fibrosis [81]. HGF interferes with TGF‐β activity primarily by reducing TGF‐β expression [79]. In the kidney, TGF‐β1 mRNA levels have been shown to be reduced with the presence of HGF, thereby preventing fibrogenic activity [79]. Transgenic mouse studies have shown that overexpression of HGF induced a marked reduction in TGF‐β1 levels and a corresponding decrease in renal fibrosis [79]. The ability of HGF to induce such downregulation is a critical component in its antifibrogenic action [82]. TGF‐β signaling was also prevented by HGF blocking the phosphorylation and nuclear translocation of Smad protein, inhibiting TGF‐β signal transduction [78].

TGF‐β was also found to have a reciprocal relationship with HGF during the progression of chronic renal failure [83]. As the chronic injury progressed, HGF expression initially seemed upregulated as a compensatory measure to combat the injury [83]. However, worsening conditions led to the increase of TGF‐β along with a subsequently suppressed HGF expression [84]. In addition, HGF appeared to serve a protective role in tubular apoptosis [85]. In an ICGN mouse model of glomerulonephritis, HGF administration significantly reduced TGF‐β expression and tubular apoptosis while enhancing tubular proliferation and regeneration [85]. These results were also reflected in acute renal failure models [83]. When mice were treated with nephrotoxic drugs such as cisplatin, HGF administration was shown to prevent histological destruction of renal tubules, suggesting the potency of HGF in both acute and chronic cases [83].

Additionally, along with TGF‐β1 activity being suppressed, collagen Type I and IV mRNA expression was shown to be decreased, and an increase in expression of MMP, specifically MMP‐2 and MMP‐9, was noted [86]. This occurred along with a decrease in the expression of tissue inhibitors of metalloproteinases (TIMPs), which led to an enhanced level of ECM degradation [86]. The presence of HGF also showcased the preservation of the epithelial phenotype of renal cells, inhibiting a transition to *α*‐SMA–positive myofibroblasts, a critical process in the development of fibrosis. The prevention of epithelial‐to‐mesenchymal transition (EMT) allows for the preservation of normal kidney structure and function [86].

While HGF was observed to hold antifibrotic effects in mouse models, its relevance to humans remains unclear. One study focused on utilizing cultured human renal fibroblasts. This revealed recombinant HGF’s ability to suppress TGF‐B1 mRNA expression and collagen III synthesis, supporting the antifibrogenic property of HGF in human renal cells [87]. Although dosing and routes of administration pertaining to HGF in human renal fibrosis are still under investigation, there are studies that reflect its impact on patients with fulminant hepatitis. Ido et al. conducted a study that revolves around recombinant human HGF (rh‐HGF) in patients with fulminant hepatitis or late‐onset hepatic failure [88]. Here, rh‐HGF was administered intravenously at a dose of 0.6 mg/m2/day for 12–14 days [88]. The results showed that rh‐HGF held no significant impact on fulminant hepatitis [88]. However, no severe renal toxicity or adverse effect was notated aside from blood pressure reduction during infusion [88]. Ido et al. drew the conclusion that the dose for rh‐HGF was low in comparison to preclinical studies involving mice; thus, further studies surrounding higher doses may need to be investigated [88].

### 2.3. HGF as a Potential Target for Treating Renal Fibrosis

#### 2.3.1. Stability and Efficacy of HGF Treatments in Renal Fibrosis Progression in a Preclinical Setting

Liu et al. were already able to show that high HGF levels in early stages of glomerulonephritis decrease as the fibrosis progresses, suggesting that endogenous HGF is consumed during the repair process [86]. Thus, HGF treatment exogenously would potentially maintain therapeutic levels [86]. In addition, when exogenous HGF was administered, such as in Matsumoto et al.’s study, the therapeutic effects were clear: Renal function was stabilized and fibrosis markers such as TGF‐β and collagen deposition were significantly reduced, an effect that was evident over the course of several weeks, suggesting a promising level of stability and efficacy [83].

Mizuno et al. emphasized the role of HGF in compensatory renal growth [89]. The administration of recombinant HGF to nephrotic mice enhanced tubular proliferation and reduced fibrotic changes, suggesting that HGF supports compensatory mechanisms in the kidney [89]. This compensatory growth is crucial for maintaining renal function in the face of ongoing injury and fibrosis, further supporting the therapeutic potential of HGF in chronic renal diseases [89]. In one instance, rh‐HGF that was intravenously injected into mice demonstrated the ability of HGF to reconstruct renal tissue and promote the formation of renal tubular cells after drug‐induced kidney injury [90]. In another case, Nakasatomi et al. demonstrated that HGF promotes renal tubule formation in an in vitro system using human proximal tubular epithelial cells [91]. This effect is further enhanced by glial cell‐derived neurotropic factor (GDNF), highlighting a cooperative interaction between these signaling pathways [91]. These findings suggest an important role for endothelial–epithelial signaling in kidney development and repair and support further investigation into these mechanisms in human systems [91].

Mori et al. showed that continuous administration of HGF has significantly improved glomerular repair and function in a progressive glomerulonephritis mode [92]. By utilizing osmotic pumps to deliver rh‐HGF across a prolonged period, stable and sustained HGF levels were able to be maintained, leading to significant improvements in glomerular repair and function [92]. Interestingly, HGF led to marked endothelial cell proliferation, which subsequently led to improved renal perfusion and function, showcasing a specific mechanism as to how HGF can promote renal recovery in fibrotic conditions [92]. In addition, inflammatory cell infiltration was reduced in HGF‐treated kidneys, decreasing the presence of macrophages and other immune cells, stabilizing the renal environment [92]. In comparison to VEGF, HGF appeared to have a broader spectrum of actions, including antifibrotic, antiapoptotic, and regenerative effects [92].

Studies observed by Gong et al. on the continuous HGF administration in rats with remnant kidneys showcase the stability of HGF activity [93]. Continuous delivery of HGF showed significant amelioration of renal dysfunction and fibrosis, highlighting the importance of the necessity to maintain consistent HGF levels for optimal therapeutic outcomes [93]. Continuous HGF administration resulted in a stable GFR and reduced proteinuria, while also inducing an increase in MMP activity and decrease in TIMP activity to create a favorable environment for ECM breakdown [94].

Yang, et al. investigated the therapeutic potential of the HGF gene in ameliorating chronic renal fibrosis [95]. Their study demonstrated that systemic administration of a naked plasmid encoding HGF significantly reduced renal interstitial fibrosis in a mouse model induced by UUO [95]. The findings revealed that exogenous HGF expression led to sustained activation of extracellular signal‐regulated kinases 1 and 2 (Erk1/2), which are key signaling molecules in renal epithelial cells [95].

Matsumoto and Nakamura explored the renotropic and antifibrogenic properties of HGF in various experimental models of renal disease [83]. They found that prophylactic administration of HGF in mice subjected to nephrotoxic agents like cisplatin, HgCl2, cyclosporine A, and tacrolimus prevented histological destruction of renal tubules and suppressed the onset of acute renal dysfunction [83]. In models of chronic allograft nephropathy, HGF administration post‐transplantation prevented early injury, reduced macrophage infiltration, and inhibited TGF‐β1 overexpression, ultimately leading to the survival of all HGF‐treated animals without later development of renal fibrosis [83].

Overall, preclinical findings show strong evidence of antifibrotic and regenerative effects of HGF. HGF has shown to induce compensatory renal growth and tubular proliferation, improved renal function, and modulation of ECM remodeling in preclinical studies. Although these findings support a strong rationale for HGF‐based therapies in the setting for renal fibrosis, human clinical studies are needed to determine safe and effective therapies.

#### 2.3.2. Translational Potential of HGF in Renal Fibrosis

Diabetes‐induced kidney fibrosis is thought to be a complex process driven by hyperglycemia that leads to increased glucose reabsorption via sodium–glucose co‐transporter‐2 [96]. This results in decreased solute delivery to the macula densa, which activates RAAS and leads to increased glomerular perfusion and subsequent renal structural changes [23]. Early glomerular injury in the setting of type 2 diabetes is thought to initially affect podocytes [97]. These differentiated cells have been shown to be involved with renal fibrosis due to injury resulting in impaired kidney function and accumulation of ECM [98]. This is specifically observed through type II diabetes mellitus, a major contributor to renal fibrosis [99].

Type II diabetes has been shown to directly alter kidney dysfunction, specifically through podocyte detachment and decrease in density [100] through hyperglycemia‐induced podocyte apoptosis [101]. Diabetes negatively impacts podocyte functionality, leading to albuminuria and low GFR [102]. Additionally, patients with diabetic nephropathy and other podocytopathies have been shown to have increased profibrotic cytokines, such as TGF‐β and VEGF [103]. Interestingly, HGF plays a protective role with podocytes by preserving nephrin and WT‐1, important factors in podocyte slit filtration [104]. Additionally, HGF has been shown to improve podocyte autophagy, a protective mechanism that removes defective proteins that are lost in diabetic nephropathy [105].

Additionally, HGF treatment inhibited the expression of α‐SMA and reduced the accumulation of collagen I and fibronectin, major components of ECM [95]. Furthermore, HGF suppressed the renal expression of the profibrogenic cytokine TGF‐β1 and its type I receptor, highlighting its potential to disrupt fibrogenic pathways and provide evidence for a possible therapeutic approach for chronic renal fibrosis [95].

Kitamura et al. explored the role of HGF in mitigating renal fibrosis by focusing on its effects on oxidative stress and proinflammatory cytokine expression [106]. Their findings revealed that HGF significantly reduced oxidative stress markers and inhibited the expression of proinflammatory cytokines, which are critical drivers of fibrosis [106]. In a murine model, the combined therapy of HGF and angiotensin receptor blockers (ARBs) resulted in a synergistic attenuation of renal fibrotic lesions compared to either treatment alone [83]. This synergistic effect was attributed to HGF’s ability to enhance the degradation of ECM and suppress the profibrotic actions of TGF‐β1 [83]. Moreover, HGF treatment led to a marked decrease in myofibroblast activation and collagen deposition, key indicators of fibrosis progression [107]. These results underscore the potential of HGF not only as a standalone therapeutic agent but also in combination therapies to effectively treat CKD [107].

Evidence supporting the positive impact on renal fibrosis patients was also investigated in human studies. A study conducted by Kellenberger et al. showed an inverse relationship between HGF and fibrosis score in protocol biopsies 1 year post renal transplant [108]. Interestingly, HGF did not reliably predict fibrosis progression or graft survival. These findings are consistent with the antifibrotic activity demonstrated in the preclinical animal studies in previous sections. As this was an observational study, a clinical trial is needed to evaluate the efficacy of HGF‐based therapies and further define clinical utility in patients with renal fibrosis.

One such clinical study conducted by Vincenti et al. looked at Phase 3 trial of the GIFT trial and showed that there were no significant differences between ANG‐3777, an HGF mimetic, and the placebo group in estimated GFR and duration of dialysis [109]. Although the GIFT trial did not meet its primary endpoint of estimated GFR, showing no significant difference between the ANG‐3777 group and placebo, there was a noted decrease in graft failure rates in the ANG‐3777 group as well as an overall favorable safety profile [109]. Furthermore, this trial targeted acute kidney injury and did not explore the effect on renal fibrosis. While the findings could not establish sufficient clinical efficiency, they suggest that HGF has a beneficial impact on graft preservation.

The failure of the GIFT trial to produce clinically relevant results suggests that the drug may not have adequately activated the pathway. ANG‐3777 is a small molecule HGF mimetic, and the GIFT trial did not include any pharmacodynamic markers, making it difficult to discern whether the drug reached its intended target in the kidney [110].

Moreover, there are safety concerns, which increase with the chronicity of the treatment. The short 3‐day course of HGF mimetic therapy in the trial appeared to be safe for participants. However, in order to treat chronic renal fibrosis, a much longer duration of months to potentially years would be required. This timeframe poses an oncogenic risk in humans due to the c‐Met protooncogene pathway not being well characterized in humans. In addition, the short course of therapy may have been insufficient to maintain the regenerative and antifibrotic effects observed in preclinical studies where HGF was given for a duration of two to 4 weeks. Future progress will depend on resolving whether the failure of the GIFT trial to meet its primary endpoint reflects a drug‐specific problem or a disease context problem.

An interesting comparison of the results from the GIFT trial can be made when looking at other studies investigating the timing of HGF administration and its outcomes on renal diseases [111]. Yang and Liu investigated the effectiveness of delayed HGF treatments administered 3 days after renal fibrosis due to UUO and found that HGF slowed the accumulation of myofibroblast and collagen deposition but did not reverse these processes [111].

Interestingly, in the GIFT trial, ANG‐3777 was administered after kidney dysfunction occurred following a kidney transplant, which yielded no significant improvements in renal function compared to the control group [109]. These findings show that while HGF treatments show potential preventive benefit and therapeutic benefit as well, full extension of this may be reduced depending on the timing of administration of HGF after renal insult [109].

## 3. Conclusion

Overall, HGF is observed to be a pleiotropic agent that holds the ability to serve as an antiapoptotic agent, anti‐inflammatory agent, antifibrotic agent, and an inflammatory biomarker. When it comes to renal fibrosis, HGF has a multifaceted role in tackling this condition through mechanisms such as TGF‐β inhibition, ECM breakdown, and endothelial repair. While it does show promising results through multiple studies, there are limitations surrounding its delivery, stability, and optimal dosing to maximize therapeutic effects in clinical settings. Studies discussed in this study used mainly animal models to explore the antifibrotic properties of HGF. The lack of human clinical trials dedicated to studying the preventive and therapeutic antifibrotic properties of HGF may limit the clinical application of our findings. Thus, a future direction would be to investigate clinical studies that use HGF on markers of renal fibrosis in human subjects.

It is also interesting to note the role of HGF in other chronic diseases associated with renal fibrosis, such as diabetes and hypertension. While HGF is shown to have protective factors against renal fibrosis, HGF serves as a strong biomarker when it comes to diabetes. Overexpression of HGF in the body has been observed to be associated with the onset of insulin resistance, which can increase the risk of developing diabetes and hypertension. Thus, it is imperative to weigh the benefits and costs of using HGF as a potential treatment for renal fibrosis. Future research is necessary to study different dosages of HGF and its impact on treating renal fibrosis and discouraging the onset of diabetes and hypertension.

An important consideration in the therapeutic use of HGF is the need to balance its antifibrotic and regenerative properties against the oncogenic potential associated with activation of the HGF/c‐Met pathway. Physiologically, transient HGF/c‐Met signaling promotes tissue repair, epithelial regeneration, angiogenesis, and cellular survival; however, persistent or dysregulated activation of this pathway has been implicated in tumor initiation, progression, and treatment resistance. HGF/c‐Met signaling has been shown to promote renal carcinoma through upregulation of circ‐CCDC66, a circular RNA that supports maintenance and expansion of cancer stem cells [112]. Additionally, deregulation and overexpression of this signaling axis have been implicated in resistance to targeted therapies through bypass activation of multiple pro‐oncogenic pathways, including Akt–mTOR, EGFR, and RAS–RAF–MEK signaling [113]. Furthermore, constitutive activation of HGF/c‐Met signaling has been associated with more aggressive tumor behavior and poorer clinical outcomes across multiple malignancies [114].

While these findings raise oncogenic risk concerns, early clinical trials of HGF‐based therapies have not shown a clear connection to causing cancer. Both the dose–response relationship and long‐term safety remain uncertain. To address this, future studies must focus on enhancing the delivery strategy through targeted, tissue‐specific methods, optimizing the dosing regimen, and continuing surveillance to identify any further risks associated with the HGF treatment. Overall, while HGF‐based therapies may raise concerns regarding tumor promotion, it is important to note that HGF activation helps with the healing and regeneration process. Finding the ideal balance and proving safety will require dedicated, long‐term patient monitoring [115].

Although HGF demonstrated promising antifibrotic properties in preclinical and observational human studies, early clinical trials have yielded mixed results, warranting further investigation to optimize therapeutic application while minimizing potential adverse effects. Special consideration in future translational studies must account for the broader effects of HGF’s role beyond renal fibrosis, such as its complex role in metabolic disorders such as diabetes and hypertension, as well as its oncogenic potential. These considerations are imperative in balancing antifibrotic benefits of HGF with long‐term safety and potential adverse effects on overall patient health.

## Author Contributions

A.F. led the literature review, prepared the initial manuscript, and conducted revisions on the manuscript. J.K. contributed to the literature review and assisted in writing the manuscript.A‐A.B. revised the manuscript and added sections. M.A. supervised the entire process and provided critical feedback.

## Funding

No funding was received for this research.

## Disclosure

All authors have reviewed and approved the final version of the manuscript.

## Conflicts of Interest

The authors declare no conflicts of interest.

## Data Availability

Data sharing is not applicable to this article as no datasets were generated or analyzed during the current study.
